# Supplementary material for: The interactome of intact mitochondria by cross-linking mass spectrometry provides evidence for coexisting respiratory supercomplexes
Source: Mol Cell Proteomics. 2017 Dec 8;17(2):216–32. doi: 10.1074/mcp.RA117.000470 (PMC5795388; doi:10.1074/mcp.RA117.000470)
Supplement: Supplemental Data [file supp_RA117.000470_133922_0_supp_23978_fzffwf.zip › spectra_annotation/mito_DR_spectra_annotation/154-1-14-1-4-1.pdf]

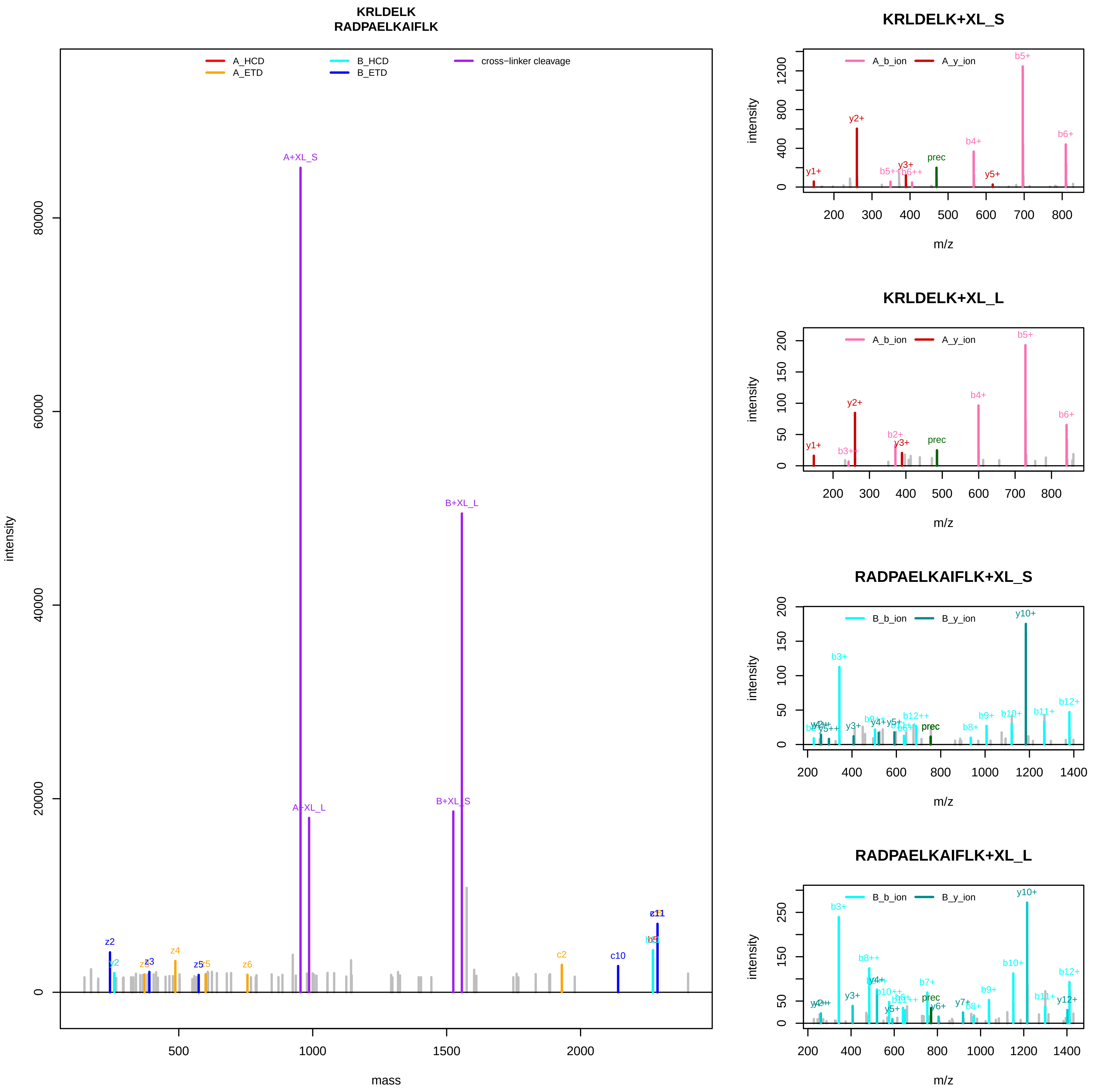
KRLDELK
RADPAELKAIFLK
A_HCD
A_ETD
B_HCD
B_ETD
cross−linker cleavage
A+XL_S
B+XL_L
A+XL_L
B+XL_S
intensity
mass
0
20000
40000
60000
80000
500
1000
1500
2000
z2
y2
z3
z4
z5
z6
c2
c10
c11
KRLDELK+XL_S
A_b_ion
A_y_ion
y1+
y2+
y3+
y5+
b4+
b5+
b6+
prec
KRLDELK+XL_L
b2+
b3++
RADPAELKAIFLK+XL_S
B_b_ion
B_y_ion
b3+
y3+
y10+
b8+
b9+
b10+
b11+
b12+
b12++
RADPAELKAIFLK+XL_L
b8++
b7+
y6+
y7+
y12+
m/z
